# Sleep disorders as a prospective intervention target to prevent drug relapse

**DOI:** 10.3389/fpubh.2022.1102115

**Published:** 2023-01-04

**Authors:** Chao Sun, Xiaojun Wang, Xuetong Huang, Yongcong Shao, Anna Ling, Huanhuan Qi, Zhuolin Zhang

**Affiliations:** ^1^School of Psychology, Beijing Sport University, Beijing, China; ^2^China Wushu School, Beijing Sport University, Beijing, China; ^3^Beihu Road Primary School, Liuzhou, Guangxi, China

**Keywords:** sleep disorders, quality of life, relapse inclination, Health Qigong, substance users

## Abstract

**Objective:**

The high rate of relapse has become the primary obstacle of drug rehabilitation. In this study, we explored the relationship between sleep disorders and relapse inclination in substance users, as well as the potential mediating mechanisms and corresponding interventions.

**Methods:**

A total of 392 male substance users were recruited to complete the questionnaires on sleep disorders, quality of life and relapse inclination. On account of this, 60 participants with sleep disorders were randomly screened and allocated to the intervention and control groups. The former received 12 weeks of Health Qigong aimed at treating sleep disorders, whereas the latter performed their regular production work.

**Results:**

Sleep disorders had a positive effect on relapse inclination, quality of life was a potential mediator of this relationship, and 12-week Health Qigong designed to treat sleep disorders improved not only their sleep quality but also their overall quality of life, which in turn reduce the tendency to relapse.

**Conclusion:**

Current research not only explores the high-risk factors influencing relapse, but also develops customized intervention strategies, which have theoretical and practical implications for decreasing relapse and increasing abstinence.

## 1. Introduction

Drug relapse refers to the use, intake, or abuse of psychoactive substances by substance users who have undergone withdrawal treatment and rehabilitation (1). According to the most recent data in 2021, China has 1.801 million substance users, with a relapse rate of 91.4%. Relapse prevention has become a top priority in drug addiction intervention and treatment. According to Chinese law, mandatory isolation is the most important method of detoxification and relapse prevention (2). Substance users in mandatory isolation centers will receive 2 years of specialized education in detoxification, therapy, rehabilitation, and reintegration into society. Simultaneously, they will engage in necessary production activities, acquire vocational skills, gain insight into the dynamics of drug control, receive instruction in the prevention of relapse, and alter undesirable patterns of behavior. However, not much progress is made in alleviating drug cravings or preventing relapse due to this (3). In order to better the abstinence rate and decrease the relapse rate of substance users, it is imperative to explore the high-risk factors of relapse and develop specialized intervention programs.

### 1.1. Sleep disorders and relapse

Sleep disorders are the inability to get normal sleep in a suitable sleep environment. The most prominent clinical manifestations include difficulty in getting to sleep and maintaining sleep for a long time, waking up early and feeling tired after waking up, which sometimes lead to physical discomfort and even physical dysfunction (4). substance users in China are more likely to experience sleep problems than the general population. Specifically, the majority of individuals who used heroin (80.24%), methamphetamine (54.16%), or ketamine (81.98%) suffered from sleep disorders (5). Due to longer sleep latency, poorer subjective sleep quality, and less efficient sleep habits, substance users reported more frequent sleep disorders and an increased need for sleep medication than non-users (5).

The neurobiological processes underlying sleep and substance abuse are intertwined, and alterations to one can have repercussions on the other (6). Acute and chronic drug use (such as opioids, cocaine, methamphetamine, morphine, etc.) has been shown to disrupt sleep in various ways, including latency to sleep onset, sleep maintenance, and sleep quality (7–9). In turn, sleep quality was inversely associated with cravings for a range of substances, including alcohol, tobacco, opiates and marijuana during detoxification (10, 11). For example, poor sleep was linked to a higher risk of drug use in a study of 60,000 teenagers, and individuals with abnormal circadian rhythms (combined with secondary sleep disorders) were more likely to develop addiction (12). Other study using the Ecological Transient Assessment, which sent surveys to 122 people in addiction treatment once a day for 3 weeks, showed a stronger association between sleep quality and craving, indicating a potential cumulative effect of sleep disorders on craving (13). Increased sensitivity to pain, augmented negative emotions, cognitive impairment, and lack of willpower are just some of the consequences of interrupted sleep, which contribute to an increased risk of relapse (13–16). It is not difficult to draw the conclusion that sleep disorders have a positive effect on the relapse of substance users, and the more severe the sleep disorders, the greater the likelihood of relapse.

### 1.2. The mediating role of quality of life

Quality of life refers to an individual's subjective evaluations about his life according to his cultural background and value system. It is shaped by each person's unique set of priorities, aspirations, and standards (17). Quality of life is a broad multidimensional structure that can be influenced by numerous factors, one of which is the quality of sleep. Previous research has looked at the relationship between elderly people's quality of life and both subjective and objective sleep quality, as well as other related factors. The findings highlight the importance of efficiency and beliefs about sleep in relation to the quality of life in healthy older adults (18). Students with chronic insomnia have a significantly lower quality of life, as evidenced by poorer physical health, higher negative emotions, fewer leisure activities, and less housework and coursework. Furthermore, college students who participate in sleep training report significant enhancements in not only sleep quality but also overall mental health and quality of life (19). In addition, more so than demographics, smoking history, and disease severity, poor sleep is a potentially modifiable risk factor for the poor quality of life in chronic obstructive pulmonary disease patients (20). Despite the fact that there are few studies that have discussed the connection between substance users' sleep and quality of life, the pertinent scientific outcomes indirectly corroborate this association.

Quality of life is not only a critical outcome variable, but also increasingly a determinant of the prognosis of substance users (21). A systematic international review of opioid users in low–and middle-income countries has shown that opioid replacement treatment would improve participants' quality of life over time, resulting in lower addiction severity index scores (21). Further, a survey of 796 people from 21 alcohol/drug service centers in Victoria and Western Australia found that patients who had undergone improvements in their social, psychological, and physical quality of life were better able to sustain the reduction in substance use over the long term (22). In brief, as life satisfaction increases, so does withdrawal motivation (23). Consequently, sleep disorders in recovering addicts may diminish their quality of life, thereby increasing their likelihood of relapse.

### 1.3. Intervention effect of Health Qigong

A growing body of evidence supports the notion that exercise not only improves physical appearance, aids in weight loss, and increases muscle mass, but also has positive effects on mood, thinking, and other factors. However, recent studies have shown that participation in physical activity does not always yield positive outcomes (24). This might be brought on by variants in exercise's structural parameters like intensity, duration, frequency, and type. In comparison to Tai Chi and general aerobic exercise, Health Qigong has been proven more effective at enhancing sleep quality (25). Qi is the universal air, while Gong refers to practice and exercise (26). Qigong, a traditional Chinese health practice with a long and storied history, may be generally divided into internal Qigong and external Qigong (27). When engaging in internal Qigong, practitioners control their inner Qi by paying attention to their bodies and breathing, whereas external Qigong, also known as Medical Qigong, calls on qualified Qigong therapists to identify energy blockages and provide Qi to help patients' energy flow (28). Health Qigong falls under the area of internal Qigong and has the potential to serve as a complementary treatment for an array of physical and psychological disorders, such as fatigue, anxiety, and depression (29). However, the relevant study on Health Qigong and sleep disorders did not include individuals with substance use disorders. Accordingly, this study will target sleep disorders to explore whether a 12-week specialized Health Qigong intervention could improve sleep quality and reduce drug relapse.

### 1.4. The current study

In conclusion, the purpose of the current study was to investigate the relationship between sleep disorders and substance users' relapse, as well as the potential mediating mechanisms and corresponding interventions. The relapse inclination was used to predict the participants' post-treatment relapse behavior because they were all receiving mandatory isolation treatment in drug rehabilitation institutions and thus had no access to external drugs. Relapse inclination refers to the propensity of substance users to intake substances again after successful treatment. We proposed the following hypotheses in light of the literature review:

H1: Sleep disorders of substance users will be directly associated with relapse inclination.
H2: Quality of life will mediate the relationship between sleep disorders and relapse inclination.
H3: Health Qigong intervention targeting sleep disorders could effectively improve sleep quality, enhance the quality of life, and then reduce relapse inclination.

## 2. Materials and methods

### 2.1. Participants

#### 2.1.1. Cross-sectional surveys (stage 1)

A total of 420 male substance users were recruited *via* convenience sampling from Guangxi rehabilitation facilities in China. The survey was conducted through a collective test of paper questionnaires, which were distributed and collected on-site. 392 valid questionnaires were gathered at the end, for a response rate of 93.33%, after invalid questionnaires were eliminated. The age of the participants was 38.78 ± 8.30 years, and the years of drug use were 4.51 ± 1.67 years. Table 1 provides a summary of the participants' demographic characteristics.

**Table 1:** Descriptives of the sample.

**Variable**	**Cross-sectional surveys**	**Longitudinal** **interventions**	
	***N***	**Intervention group**	**Control group**
Education			
Elementary	158	11	12
Junior	218	17	17
Senior	16	1	0
Marriage			
Single	200	10	9
Married	123	7	4
Divorce	69	12	16
Drug abuse pattern			
Snorting	210	13	14
Intramuscular injection	4	1	2
Intravenous injection	111	8	6
Combination	67	7	7
Relapse			
Yes	340	24	26
No	52	5	3

#### 2.1.2. Longitudinal Health Qigong interventions (stage 2)

In accordance with the findings of the cross-sectional survey, 60 individuals with sleep disorders were randomly assigned to the intervention group or the control group. Exclusion criteria: (1) patients with a psychiatric diagnosis; (2) individuals with interpersonal communication disorders; (3) those who completed drug treatment in isolation within four months; and (4) individuals with benzodiazepine use disorders. Ultimately, each group contained 29 participants. There were no significant differences in age (36.86 ± 6.84 vs. 38.14 ± 5.76) and the years of drug use (4.69 ± 1.58 vs. 4.65 ± 1.88) between the intervention and the control group (*p* > 0.05). Additional demographic details for both groups are presented in Table 1. The drug rehabilitation center approved this study after conducting an ethics review. All individuals voluntarily participated and provided written informed consent.

### 2.2. Measures

#### 2.2.1. Pittsburgh sleep quality index

This scale is administered to evaluate the sleep quality of an individual over the past month (30). It consists of 18 items, including 7 dimensions: subjective sleep quality, time to fall asleep, sleep duration, sleep efficiency, sleep disturbance, hypnotic drugs and daytime dysfunction, each of which ranged from 0 to 3, resulting in a total score from 0 to 21. The higher the score, the more severe the sleep disturbance. There is a consensus that a PSQI score of 7 or lower indicates healthy sleep, while a score above 7 implies sleep disorders. In current study, the Cronbach's alpha was 0.74.

#### 2.2.2. The MOS item short from health survey (SF-36)

It's used to assess the composite indicator of both physiological and psychological well-being (31). This scale consists of 36 items, including eight dimensions: PF, physical functioning; RP, role physical; BP, bodily pain; GH, general health; VT, vitality; SF, social functioning; RE, role emotional; and MH, mental health. The higher the score, the better the quality of life. In current study, the Cronbach's alpha was 0.90.

#### 2.2.3. Relapse inclination questionnaire

This scale is used to determine the likelihood that participants would resume substance use after completing drug treatment (32). It evaluates five dimensions: confidence in drug withdrawal, real influence of drugs, objective environment, degree of physical and mental damage and support system. It consists of 18 items with responses graded on a six-point Likert scale (least severe = 0 to most severe = 5). Higher scores indicate greater likelihood of relapse. In this investigation, the Cronbach alpha value was 0.89.

#### 2.2.4. Intervention program and implementation of Health Qigong

From the perspective of traditional Chinese medicine, the normal sleep-wake pattern is determined by the balance of Yin and Yang. Yin deficiency and Yang excess are the primary causes of sleep disorders. Moreover, the kidney is the source of Yin and is connected directly or indirectly to all body functions. To put it another way, sleep disorders stem from a deficiency of kidney Yin (33). By engaging in specific Health Qigong movements like Deer Running and Golden Rooster Dawn, it is feasible to effectively accomplish the goals of nourishing Yin and tonifying the kidney. On the basis of this concept, we designed a set of targeted Health Qigong prescriptions, including the following actions: the ready form, Holding Sky with Hands, Shuangyu Xuange, Longdeng, Tiger Pounce, Rouji Style, Deer Running, Golden Rooster Dawn, Bird Flying, Returning Qi to the Source, and the ending form. Implementation environment: On the playground of Guangxi rehabilitation facilities. Qualifications of the intervener: It was carried out jointly by the ambassador of Health Qigong of Guangxi Province and the director of the Traditional Sports Health Research Center of the China Wushu Academy. All interventions took place from 4:00 to 5:00 p.m., four times per week, for a total of 12 weeks.

### 2.3. Procedures

In the cross-sectional questionnaire study, the PSQI, SF-36, and RIQ were administered in groups. The PSQI score was applied to screen participants with sleep disorders for the longitudinal intervention study, and 60 of them were randomly assigned to the intervention group and the control group. Besides, the questionnaire's results were taken as pre-test scores. There were no significant differences in PSQI (17.46 ± 2.32 vs. 16.78 ± 3.14), SF-36 (456.64 ± 117.36 vs. 499.85 ± 133.36) and RIQ (1.59 ± 0.51 vs. 1.32 ± 0.57) between the intervention and the control group (*p* > 0.05). Both groups then received the experimental intervention for a full 12 weeks. Whereas the control group performed their regular production work without any extra exercise, the intervention group engaged in Health Qigong. There are no other different settings. At the conclusion of the experiment, PSQI, SF-36, and RIQ were measured as the post-test score.

### 2.4. Statistical analysis

#### 2.4.1. Data from cross-sectional surveys

To begin, Harman single-factor test was used to check for common method bias because all variables were measured by questionnaires. Secondly, descriptive analysis and Pearson correlation were conducted for the main variables. Subsequently, the PROCESS plug-in is used to test the mediation model employing bias corrected percentile bootstrap CI.

#### 2.4.2. Data from longitudinal interventions

A 2 (group: intervention group, control group) ×2 (time: pre-test, post-test) two-factor mixed design analysis of variance was used to compare baseline and post-intervention scores on measures of sleep disorders, quality of life and relapse inclination between the intervention and control groups. Furthermore, correlation analysis was used to evaluate the relationships among the changes in PSQI, SF-36 and RIQ in the intervention group before and after the intervention to determine whether all variables altered to the same extent.

## 3. Results

### 3.1. Results of cross-sectional surveys

#### 3.1.1. Common method deviation test

The Harman single factor test was used to examine the common method deviation. All the questionnaire items were included in exploratory factor analysis. The first factor explained 22.51% of the total variance, which was less than the 40% threshold, indicating that there was no common method deviation.

#### 3.1.2. Descriptive statistics and correlations of variables

Based on the diagnostic criteria for sleep disorders (PSQI > 7), all participants suffered from sleep disturbances to varied degrees (*M*_PSQI_ = 15.98, *SD* = 2.32; minimum = 9, maximum = 22.50). Table 2 revealed that PSQI was negatively correlated with SF-36 (*r* = −0.41, *p* < 0.01), and positively correlated with RIQ (*r* = 0.49, *p* < 0.01). SF-36 was negatively correlated with RIQ (*r* = −0.59, *p* < 0.01). In light of this, we can dig deeper into how the aforementioned factors interact with one another.

**Table 2:** Descriptive statistics and intercorrelations among study variables.

	**M ±SD**	**PSQI**	**SF-36**	**RIQ**
PSQI	15.98 ± 2.32	1.00		
SF-36	514.15 ± 136.73	−0.41^**^	1.00	
RIQ	1.40 ± 0.69	0.49^**^	−0.59^**^	1.00

** *p* < 0.01, the same below.

**Figure 1:**
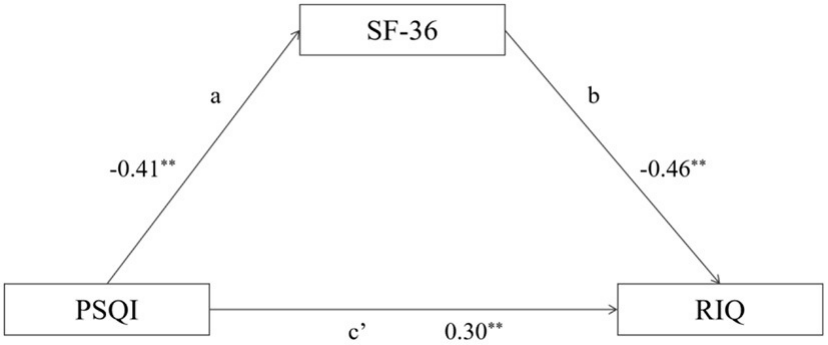
The path diagram of mediating effect of SF-36 between PSQI and RIQ.

The model of this investigation is constructed in accordance with the theoretical hypothesis and the findings of the correlation analysis (as depicted in Figure 1). Model 4 in the PROCESS of SPSS macroprogram was used to test the mediating effect of quality of life, with PSQI serving as the independent variable, SF-36 as the mediating variable, and RIQ as the dependent variable. Table 3 demonstrated that the direct impact of PSQI on RIQ is 0.089, *p* < 0.01, while the indirect impact is 0.06, *p* < 0.01. The model's mediating effect is statistically significant, and indirect effect comprised 39.09% of the total effect.

**Table 3:** The analysis results of mediating effect of SF-36.

	**Effect value**	**LLCI**	**ULCI**
Direct effect	0.0889	0.0640	0.1137
Direct effect	0.0570	0.0427	0.0725
Total effect	0.1458	0.1199	0.1717

LLCI, The lower limit of the confidence interval; ULCI, The upper limit of the confidence interval.

### 3.2. Results of longitudinal interventions

**Figure 2:**
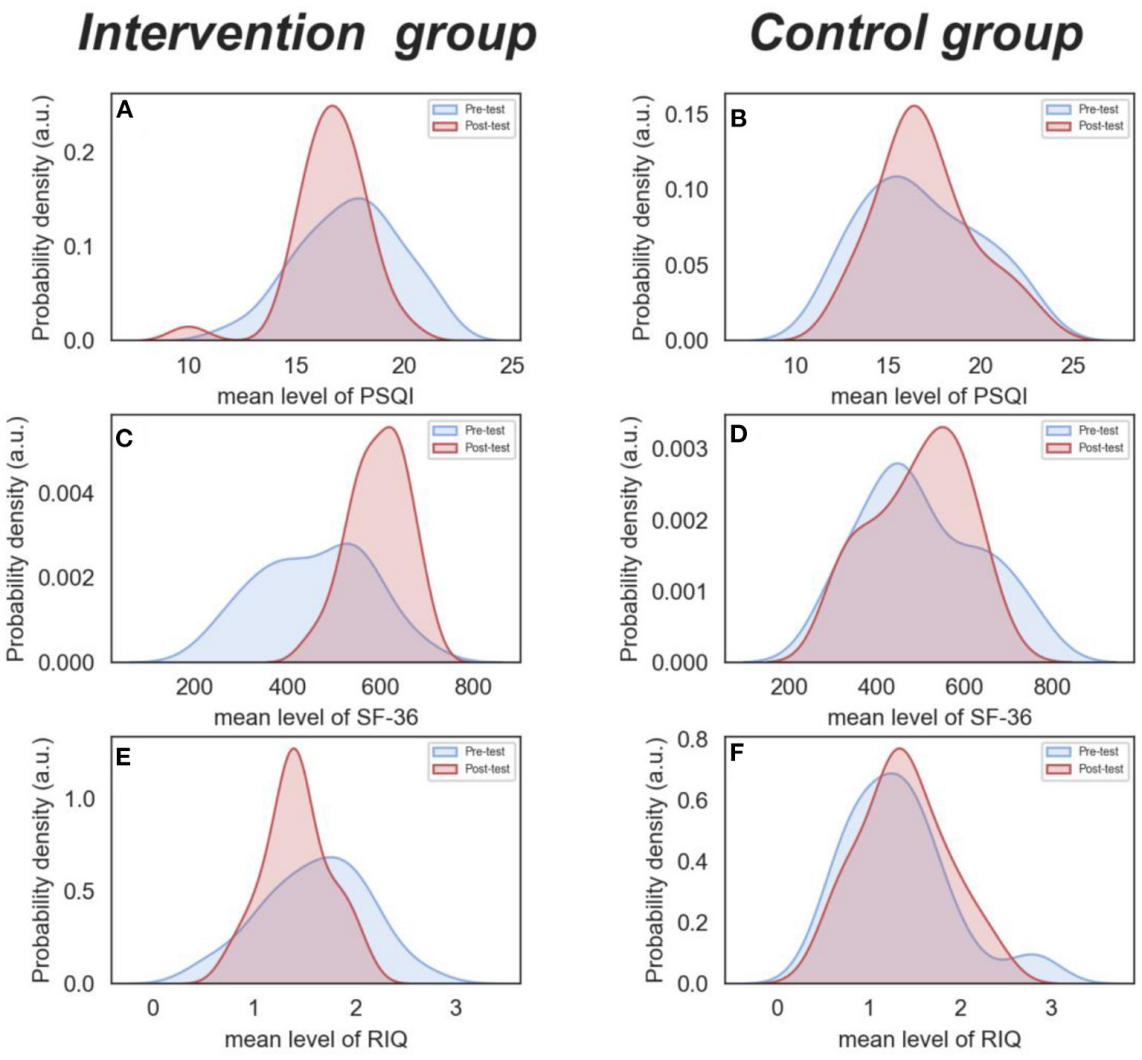
**(A–F)** Pre-test and post-test comparison of PSQI, SF-36, and RIQ between the two groups.

Using two-factor mixed design ANOVA, it was discovered that there was a statistically significant interaction between group and time in PSQI score (*F* = 8.05, *p* = 0.006, *partial* η2 = 0.126). The main effect of group (*F* = 0.04, *p* = 0.852, *partial* η^2^ = 0.001) and time (*F* = 2.48, *p* = 0.121, *partial* η^2^ = 0.042) were not significant. The simple effect analysis found that the pre-test scores (17.46 ± 2.32) of the intervention group were higher than the post-test scores (16.58 ± 1.76), while the control group's scores remained relatively stable (16.78 ± 3.14 vs. 17.03 ± 2.59) (see Figures 2A, B).

In terms of the SF-36 score, the interaction between group and time was statistically significant (*F* = 37.14, *p* < 0.001, *partial* η^2^ = 0.399). The main effect of group (*F* = 1.22, *p* = 0.274, *partial* η^2^ = 0.021) was not statistically significant. However, the main effect of time was significant (*F* = 31.66, *p* < 0.001, *partial* η^2^ = 0.361). The simple effect analysis revealed that the pre-test scores (456.64 ± 117.36) of the intervention group was lower than that of the post-test (594.55 ± 61.32), while the difference of the control group was not statistically significant (499.85 ± 133.36 vs. 494.36 ± 117.36) (see Figures 2C, D).

With regard to RIQ score, the interaction between group and time was statistically significant (*F* = 8.08, *p* = 0.006, *partial* η^2^ = 0.126). Nevertheless, the main effect of group (*F* = 1.77, *p* = 0.189, *partial* η^2^ = 0.031) and time (*F* = 2.22, *p* = 0.142, *partial* η^2^ = *0*.038) were not significant. The simple effect analysis found that the pre-test scores (1.59 ± 0.51) of the intervention group were higher than the post-test scores (1.41 ± 0.32). However, the outcomes for the control group stayed relatively constant (16.78 ± 3.14 vs. 17.03 ± 2.59) (see Figures 2E, F).

The difference scores, which were calculated by subtracting the corresponding pretest scores from the posttest scores of PSQI, SF-36 and RIQ, were indicated as DPSQI, DSF-36, and DRIQ, respectively. Correlation analysis revealed that DPSQI was negatively correlated with DSF-36 (*r* = −0.31, *p* < 0.05), and positively correlated with DRIQ (*r* = 0.57, *p* < 0.01). DSF-36 was negatively correlated with DRIQ (*r* = −0.42, *p* < 0.01).

## 4. Discussion

### 4.1. Sleep disorders and relapse

Consistent with previous research, sleep disorders positively predicted relapse inclination in substance users, which supported hypothesis 1. Sleep disorders have been linked to an increase in amphetamine's sensitization (34), a enhance in conditioned place preference for methamphetamine (35), and a rise in the speed with which one learns to self-administer cocaine and respond on a progressive ratio schedule (36). Furthermore, the fragmentation of rapid-eye-movement sleep speeds up the emergence of cocaine cravings (37). Sleep disorders have a significant impact on dopamine-mediated mesolimbic circuits, which are also in charge of the regulation of reward. Sleep deprivation decreases the availability of D2/D3 dopamine receptors in the ventral striatum (38, 39), which increases the risk of risk-taking behavior and compulsive drug use (40, 41). Consequently, sleep disorders in the context of substance use are a profound research focus, and identifying and treating sleep disorders may be a crucial measure to prevent substance abuse and prevent relapse.

### 4.2. The mediating role of quality of life

This study further explored how sleep disorders contributed to relapse inclination and discovered that quality of life might be a mediator of this relationship, supporting hypothesis 2. The first path, wherein sleep disorders were negatively related to quality of life, was in line with previous research. According to the four-dimensional model of quality of life, there are four aspects of quality of life: physical health, mental health, social health, and spiritual health (42). In terms of physical health, sleep disorders are connected with dysfunction in the majority of body systems, such as nervous system disorders, higher cortical, endocrine and metabolic (43). When it comes to mental health, sleep disorders could cause or exacerbate a host of psychological disorders, including impulse control, behavioral suppression, and addiction (44). As for social health, sleep disorders were associated with increased interpersonal stress and worse evaluations of interpersonal effectiveness (45, 46). One more downside of poor sleep is a diminished capacity for empathy and connection with others (47). With regard to spiritual health, Muslim women's quality of sleep is closely related to their spiritual wellbeing and religious practice (48).

The second path of the mediation model, wherein quality of life was negatively related to relapse inclination, was supported by relevant studies. After implementing a “community intensification” strategy to enhance the social and environmental quality of life for alcohol-dependent individuals, Hunter and Azrin discovered that participants had high rates of abstinence (49). The decline in substance abuse may be attributable to the rise in satisfaction brought on by the enhancements in living conditions. The self-medication hypothesis postulated substance users might take substance abuse as a coping mechanism to manage psychological stress, pain, or severe/complex mental problems. Once they discovered alternatives to enhance their quality of life, they were more likely to give up drugs (50).

### 4.3. Intervention effect of Health Qigong

In stage 2, we proved that Health Qigong intervention targeting sleep disorders could successfully enhance sleep quality, raise quality of life, as well as reduce the likelihood of relapse, thereby confirming hypothesis 3. Health Qigong for 12 weeks has been consistently confirmed to improve various aspects of sleep, including latency to sleep onset, total sleep time, efficiency of sleep, and daytime dysfunction, among others (51, 52). Health Qigong, a mind-body exercise that emphasizes meditation and deep breathing, improves respiratory function and regulates all of the body's organs through relaxation (25). This may increase melatonin (53), reduce inflammation, and affect the stress response pathways (54), which will lessen sleep disorders. Healthy sleep can not only aid in the recovery from physical fatigue, but also make a significant and valuable contribution to the development of mental health. Accordingly, it stands to reason that better life quality will follow from fewer sleep disorders. Furthermore, addiction is merely a means of pursuing joy and happiness, and the allure of addiction can be diminished by leading a more fulfilling life (55). To put it another way, individuals are less likely to relapse if they are more content with their lives.

## 5. Conclusion

The current study identified sleep disorders is closely related to the relapse of substance users, and quality of life was a potential mediator of this relationship. In addition, a 12-week Health Qigong program designed to treat sleep disorders improves not only sleep quality but also the overall quality of life, which in turn reduces the tendency to relapse.

## Data availability statement

The raw data supporting the conclusions of this article will be made available by the authors, without undue reservation.

## Ethics statement

The studies involving human participants were reviewed and approved by Ethics Committee of the School of Psychology at the Beijing Sport University. The patients/participants provided their written informed consent to participate in this study.

## Author contributions

The intervention of Health Qigong was carried out by XW and AL. CS performed data analysis and carried out the bulk of the literature review and manuscript writing. XW and YS played an editorial role when it came to writing up the research study. Material preparation and data collection were performed by all authors. All authors contributed to the study conception and design and read and approved the final manuscript.
