# Supplementary material for: Sleep disorders as a prospective intervention target to prevent drug relapse
Source: Front Public Health. 2023 Jan 4;10:1102115. doi: 10.3389/fpubh.2022.1102115 (PMC9846318; doi:10.3389/fpubh.2022.1102115)
Supplement: Supplementary file 1 [file Table_1.DOCX]

Supplementary Material

# 1 Relapse Inclination Questionnaire

1. I am well prepared for the many setbacks and difficulties I will encounter after my dissolution.

0. very adequate 1. very adequate 2. relatively adequate

3. adequate 4. very inadequate 5. not prepared at all

2. I believe that addiction can be kicked.

0. completely possible 1. very likely 2. likely

3. unlikely 4. almost impossible 5. absolutely impossible

3. This time I was determined not to return to smoking.

0. very determined 1. very big 2. less

3. very small 4. very small 5. not determined

4. Now I am on drugs.

0. strong hatred of disgust 1. detest 2. indifferent

3. eagerly 4. very eager 5. strongly desire

5. I am confident that I will not touch drugs again after my release.

0. completely sure 1. very sure 2. somewhat sure

3. a little unsure 4. very unsure 5. Not at all sure

6. I believe I can guarantee that I will be drug-free for at least _______ after my dissolution

0. 3,5 years 1. one year 2. half a year

3. two months 4. one month 5. one week

7. I wish I could get back on drugs and have a good time as soon as I got out of prison.

0. not longing 1. eager 2. more eager

3. very eager 4. very eager 5. extremely eager

8. After detoxification, when faced with the temptation of a drug addict friend, I will.

0. very strongly resist 1. resist strongly 2. resisted somewhat

3. resisted 4. swayed 5. relapse

9. I consider my willpower to be.

0. very strong 1. very strong 2. relatively strong

3. relatively weak 4. very weak 5. very weak

10. Because of chronic drug use, my physical health.

0. very good 1. very good 2. relatively good

3. relatively poor 4. very bad 5. very bad

11. My family and friends and relatives supported and encouraged me to get clean.

0. strongly support 1. positively support 2. More supportive

3. Support 4. not very supportive 5. Fully support

12. My mind would unconsciously pop up with scenes of past drug use or drug friends or dealers.

0. never 1. ever1 or2 times 2.1 or2 months/time

3. 1 time/week 4. 2 or 3 times/week 5. 1,2 times/day

13. Post-dissolution for drugs I will.

0. Never touch 1. basically don't touch

2. may try a little 3. less than in the past

4. about the same as in the past 5. more than in the past

14. After I leave the facility, if I want to use drugs, to get them is.

0. almost impossible 1. extremely difficult 2. very difficult

3. not difficult 4. very easy 5. very easy

15. After I am released, my former drug friends will persuade me to return to drugs.

0. absolutely impossible 1. almost impossible 2. unlikely

3. likely 4. very likely 5. definitely possible

16. After I am released, I will move to a new place where no one knows me in order to get rid of my drug friends and drugs once and for all.

0. definitely possible 1. very likely 2. Possibly

3. unlikely 4. almost impossible 5. absolutely impossible

17. Because of chronic drug use, the drugs have had an impact on my mental and willpower.

0. No effect at all 1. little to no harm 2. Some harm

3. seriously hurt 4. almost collapsed 5. completely destroyed

18. After the dissolution, the drug dealer I used to know will offer to supply me with drugs.

0. absolutely impossible 1. almost impossible 2. very unlikely

3. likely 4. very likely 5. definitely possible

# 2 The MOS Item Short from Health Survey

1. In general, your health condition is.

①Very good ②Very good ③Good ④Ordinary ⑤poor

2. How do you feel about your health now compared to a year ago?

①Better than1 year ago ②Better than1 year ago

③About the same as1 year ago ④Less than1 year ago

⑤ Much worse than1 year ago

3. The following problems are related to daily activities. Please think about whether your health condition limits these activities. If there are limitations, to what extent?

A. Health condition that limits heavy physical activity? Such as running and lifting weights, participating in strenuous sports, etc..

①Large restrictions ②Some restrictions ③No restrictions at all

B. Health conditions that limit moderate activity. Such as moving a table, sweeping the floor, playing tai chi, doing simple gymnastics, etc..

①Large restrictions ②Some restrictions ③No restrictions at all

C. Health condition limits handheld daily necessities? Grocery shopping, shopping, etc..

①Large restrictions ②Some restrictions ③No restrictions at all

D. health condition limits the number of flights of stairs up.

①Large restrictions ②Some restrictions ③No restrictions at all

E. health condition limits the upper stairs to.

①Large restrictions ②Some restrictions ③No restrictions at all

F. Health conditions that limit bending, stooping, squatting.

①Large restrictions ②Some restrictions ③No restrictions at all

G. Health conditions that limit walking more than1500 meters.

①Large restrictions ②Some restrictions ③No restrictions at all

H. Health conditions limiting the distance walked to1000 m.

①Large restrictions ②Some restrictions ③No restrictions at all

I. Health conditions limiting the distance walked to100 meters.

①Large restrictions ②Some restrictions ③No restrictions at all

J. Health conditions that limit their ability to bathe and dress themselves.

①Large restrictions ②Some restrictions ③No restrictions at all

4. During the past4 weeks, have you had any of the following problems with your work and daily activities because of your physical health?

A. Reduced time for work or other activities because of physical health problems:

① Yes ②No

B. What I wanted to do because of health problems can only be partially completed:

① Yes ②No

C. Restrictions on the kind of work or activity you want to do because of physical health problems:

① Yes ②No

D. Increased difficulty completing work or other activities because of physical health problems (e.g., requiring extra effort):

① Yes ②No

5. During the past4 weeks, have you had any of the following problems in your work and daily activities due to emotional reasons (such as depression or worries)?

A. Reduced work or activity time because of emotions:

① Yes ②No

B. Because what the emotion would have wanted to do could only be partially accomplished: ① Yes ②No

C. Because emotions do things less carefully than usual:

① Yes ②No

6. In the past4 weeks, to what extent has your poor health or mood affected your normal social interactions with family, friends, neighbors or groups?

①No effect at all ②A little influence

③Moderate impact ④High impact

⑤Very high impact

7. In the past4 weeks, have you had any physical pain?

①No pain at all ②A little pain

③Moderate pain ④Severe pain

⑤Very severe pain

8. Did your physical pain affect your work and household chores in the past4 weeks?

①No effect at all ②A little influence

③Moderate impact ④High impact

⑤Very high impact

9. The following questions are about your own feelings in the past1 month, what is your situation for each of the things mentioned in the questions?

A. Do you find life fulfilling.

①All the time full ②Most of the time full

③More time to fill ④Part of the time is full

⑤A small part of the time is full ⑥No such feeling

B. Are you a sensitive person.

①Sensitive all of the time ②Sensitive most of the time

③More time-sensitive ④Partly time-sensitive

⑤Sensitive a small amount of time ⑥No such feeling

C. You are in a very bad mood and nothing can cheer you up:.

①All the time ②Most of the time

③More of the time ④Some of the time

⑤Some of the time this is the case ⑥No such feeling

D. You are mentally at peace with.

①All the time ②Most of the time

③More of the time ④Some of the time

⑤Some of the time this is the case ⑥No such feeling

E. You do things with energy.

①All the time ②Most of the time

③More of the time ④Some of the time

⑤Some of the time this is the case ⑥No such feeling

F. Your depressed mood.

①All the time ②Most of the time

③More of the time ④Some of the time

⑤Some of the time this is the case ⑥No such feeling

G. you feel exhausted.

①All the time ②Most of the time

③More of the time ④Part of the time is like this

⑤Some of the time this is the case ⑥No such feeling

H. You are a happy person:.

①All the time ②Most of the time

③More of the time ④Some of the time

⑤Some of the time this is the case ⑥No such feeling

I. You feel bored with.

①All the time ②Most of the time

③More of the time ④Some of the time

⑤Some of the time this is the case ⑥No such feeling

10. Unhealthy affects your social activities (such as visiting friends and relatives).

①All the time ②Most of the time

③More of the time ④Some of the time

⑤Some of the time this is the case ⑥No such feeling

11. Please see each of the following questions, which answer best fits your situation?

A. I seem to get sick easier than others, right?

①Absolutely correct ②Mostly correct

③Can't be sure ④Mostly wrong

⑤ Absolute error

B. I'm as healthy as anyone around me, right?

①Absolutely correct ②Mostly correct

③Can't be sure ④Mostly wrong

⑤ Absolute error

C. I think my health is getting worse, right?

①Absolutely correct ②Mostly correct

③Can't be sure ④Mostly wrong

⑤ Absolute error

D. My health is very good, right?

①Absolutely correct ②Mostly correct

③Can't be sure ④Mostly wrong

⑤ Absolute error

# 3 Pittsburgh Sleep Quality Index Scale

1. The time you usually go to bed in the past month? The time to go to bed is _______ (HH:MM)

2. How long (minutes) did it usually take you to fall asleep each night in the past month? ________ minutes

3. What time do you usually get up every morning in the past month? Wake up time _______ (HH:MM)

4. How much sleep did you actually get each night in the past month? Actual hours of sleep per night ______

*Choose one answer from each of the following questions that best fits your situation and check it.*

5. In the past month have you often had trouble sleeping because of the following problems

(a) Inability to fall asleep within 30 minutes

A. None B. <1 time/week C.1 to2 times/week D. ≥3 times/week

(b) Easy or early awakening at night

A. None B. <1 time/week C.1 to2 times/week D. ≥3 times/week

(c) Getting up at night to go to the toilet

A. None B. <1 time/week C.1 to2 times/week D. ≥3 times/week

(d) Poor breathing

A. None B. <1 time/week C.1 to2 times/week D. ≥3 times/week

(e) Loud snoring or coughing

A. None B. <1 time/week C.1 to2 times/week D. ≥3 times/week

(f) Feeling cold

A. None B. <1 time/week C.1 to2 times/week D. ≥3 times/week

(g) Feeling too hot

A. None B. <1 time/week C.1 to2 times/week D. ≥3 times/week

(h) Nightmares

A. None B. <1 time/week C.1 to2 times/week D. ≥3 times/week

(i) Presence of pain

A. None B. <1 time/week C.1 to2 times/week D. ≥3 times/week

(j) Other things that interfere with sleep

A. None B. <1 time/week C.1 to2 times/week D. ≥3 times/week

6. Your rating of total sleep quality in the past month

A. Very good B. Fairly good C. Bad D. Very bad

7. In the past month, have you often had to take medication (either from a doctor's prescription or from an outside pharmacy) to fall asleep?

A. None B. <1 time/week

C.1 to2 times/week D. ≥3 times/week

8. Have you often felt sleepy and had difficulty staying awake in the past month?

A. None B. <1 time/week C.1 to2 times/week D. ≥3 times/week

9. Have you not had enough energy to do things in the last month?

A. None B. <1 time/week C.1 to2 times/week D. ≥3 times/week
